# 3D Printed Cosmetic Covers for Lower Limb Prosthetics

**DOI:** 10.33137/cpoj.v6i2.42176

**Published:** 2023-12-22

**Authors:** K Efstathiou, A McGarry

**Affiliations:** 1 OrthoDesigns, Giannou Kranidioti, Limassol, Cyprus.; 2 Department of Biomedical engineering, University of Strathclyde, Glasgow, UK.

**Keywords:** Cosmetic Cover, Aesthetic Covers, Lower Limb, Artificial Limb, Amputation, Prosthetics, Additive Manufacturing, 3D Printing

## Abstract

Cosmetic covers provide better aesthetic appearance and may facilitate increased acceptance of the prosthesis. Traditionally, cosmetic covers aimed to achieve an aesthetic and realistic appearance; through time, a fresh perspective has developed on cosmetic covers where traditional/realistic covers evolved into a custom-made product, which truly promotes the patient's personality. The objectives of the study were to gather information from five well-known companies in the cosmetic cover industry (UNYQ, Limb-Art, Alleles, WillowWood and Aqua-Leg), analyse and compare their design elements using the Pugh matrix, and suggest a novel design using the best performing criteria of each design. The overall results of the Pugh matrix revealed the opportunity of a new design with improved design elements mainly in terms of “cover fit/aesthetics”, “ease of attachment” and “practicality”. The novel design had a vast difference in total score from the second-best design, revealing the improvement possibilities such cover design may have in the future. Although the study successfully presented a theoretical novel design, it was based on data found through literature and web resources, making the overall results of the study more objective rather than subjective. Future research is encouraged to be conducted based on a more subjective approach towards cosmetic covers.

## INTRODUCTION

Prosthetic users make use of lower limb prosthetics in their everyday life to complete tasks that they could do before their amputation. Even though a prosthesis may provide a wide variety of functional benefits, many patients choose to reject all these benefits over a poor cosmetic appearance.^1^ Therefore, the patient's body image may be a crucial aspect to consider when prescribing a prosthesis. Poor cosmetic appearance may consist of factors such as bulky interface materials; abnormal appearance of the suspension system below clothes and/or lack of anatomical symmetry.^2^ Conversely, a good cosmesis may assist prosthetic acceptance; promote functional recovery; positively affect outcome measures and improve self and social acceptance of the patient.^2^ Although cosmetic appearance constitutes a huge factor in the patients' lives, the available literature regarding cosmetic covers is limited. In the current study, major companies who produce cosmetic covers on a commercial scale were identified.

Furthermore, each company's cover design was briefly outlined, and a list of criteria was developed. Moreover, all designs were compared in a pairwise fashion based on these criteria and a theoretical novel design was then presented utilising the highest performing criteria to create an improved design.

Traditionally, cosmetic covers focused on achieving the best aesthetic appearance possible, mirroring the image of the patient's sound leg. Through time, a fresh perspective has developed on cosmetic appearance where the traditional/realistic covers have evolved into a more creative product, transforming the concept of disability into a concept of super-ability, promoting the user's personality.^3^ Studies have revealed that about 60% of prosthetic users were neutral or dissatisfied with traditional designs, resulting many companies to divert into new manufacture technologies.^4^ Companies are now able to produce custom made covers on a commercial scale, encouraging users towards personalised covers which truly reflect their personality and do not necessarily need to represent a natural look.

There are many ways to produce a lower limb cosmetic cover. Most prosthetists historically used a Plastazote foam block, which was then shaped and matched to the patient's sound leg to provide a more cosmetic appearance of the prosthesis.^2^ Although foam covers have been an affordable and lightweight option, they may be highly disadvantageous on the aspects of durability and customisation.

Technological advancements have facilitated companies to move into injection moulding techniques, to produce more durable, detailed, and water-resistant silicone covers.^5,6^ Recently, 3D printing technologies have been introduced in the industry, making cosmetic covers fully customisable, whilst affordable.

Additive manufacturing, of which the most known form is 3D printing, combines a group of emerging and innovative techniques to produce covers based on digital models using a layer-by-layer accumulation approach.^7^ The most common techniques are Multi Jet Fusion (MJF) and Selective Laser Sintering (SLS); both are Powder Bed Fusion (PBF) based techniques that use Nylon powder/Polyamide to produce the covers. The main difference between these techniques is that SLS uses a CO2 laser as a heat source, where MJF utilises an array of infrared lamps as a heat source along with a fusing agent that absorbs infrared radiation energy. MJF also uses a water-based detailing agent to inhibit the powder's fusion near the part edges, which enhances the overall cover's quality.^8^ SLS has generally been used more, as it was initially developed during the 1980s, where MJF is a more recent technique emerging in 2014. A recent study,^8^ having analysed and compared these two methods, concluded that MJF printed samples presented better printing quality and stronger bonding strength between layers; MJF samples also had higher tensile and flexural strength with a better surface finish compared to the SLS samples. Schematic illustration of the two PBF processes can be found in Cai et al., recent publlication.^8^

Manufacture of cosmetic covers has been becoming more exciting and creative over recent years, as many companies utilised these production methods. Some of the most well- known companies in the cosmetic cover industry are UNYQ, Limb-Art, Alleles, WillowWood and Aqua-Leg.

The selection procedure aimed to include companies involved in all three types of manufacture methods; foam, silicone and 3D printed. Initially, many companies that produce foam covers were identified, but WillowWood was chosen amongst them as it provided adequate information about their covers. Furthermore, Ottobock company was the most popular amongst the production of silicone covers, but there was insufficient information about their covers which resulted in choosing the next available candidate, Aqua-Leg. The rest of the companies, UNYQ, Limb-Art and Alleles, were some of the few companies who provided custom made covers and were termed as appropriate for this study. The objectives of the current study were to gather information from these five well-known companies in the cosmetic cover industry, analyse and compare their design elements using the Pugh matrix, and suggest a novel design using the best performing criteria of each design.

## METHODOLOGY

Following the analysis of the five different designs, a Pugh matrix was used to compare them with each other and produce a theoretical novel design.

The Pugh matrix is a diagram that allows to make a pairwise comparison between several designs against a set of criteria and then decide which design meets best these criteria. It also allows a degree of qualitative optimisation of the designs through the generation of new designs. One of the most important aspects of a Pugh matrix is to correctly identify the criteria, as the robustness and validity of the outcome is fundamentally dependent on an appropriate set of criteria.^9^ Criteria selection was based upon factors considered important in evidence-based literature and from websites themselves.

Cosmetic appearance and fit of the cover have been an essential factor for prosthetic users; therefore, the first and one of the most important criteria was ***“cover fit/aesthetics”***, which appraised the cover's cosmetic appearance and fit on the prosthesis. Cosmetic appearance was evaluated more towards the design's artistic and creative side and not the realistic/natural look.

As prosthetic users already have high expenses regarding the prosthesis, liners, etc., the cover's cost was an essential factor that a prosthetic user may consider before buying the cover.^2^ Consequently, the second criterion was ***“economical”*** and referred to the cover's price.

***“Lightweight”*** has been set as the third criterion and appraised the weight of each cover. The Cover's weight may be a crucial factor for some patients, as extra weight increase stresses between joint surfaces and skin.^10^

The durability of the cover was considered as an important factor, especially for active walkers.^4^ The criterion of ***“durability”*** was about the cover's robustness and strength under low/medium impacts.

The next very important criterion was ***“maintenance/repair”*** which referred to the maintenance, if any was required; the repairability of the cover, if it gets damaged; and if any warranty was available with the cover. Prosthetic users have considered maintenance, repairability and warranty as an important aspect of a cosmetic cover.^4^

Moreover, the next two criteria were ***“ease of attachment”*** and ***“practicality”***. Ease of attachment was simply referring to the attachment method and how easy it is to don and doff the cosmetic cover, which is very important from the user's perspective. Practicality was about when alignment changes were applied on the prosthesis and if the cover was still usable and cosmetically acceptable after these changes. This criterion was considered as significant, as the overall cosmetic appearance may be affected due to the low practicality of a cover.

The next criterion was ***“customisation”*** and referred to the customisation options available for each design. A variety of customisation options allowed users to choose a cover which truly reflects their personality. Therefore “customisation” was an important criterion to include.

The last five criteria were ***“ease of cleanliness”, “insurance”, “protection”, “clear website instructions” and “reliability”.*** Ease of cleanliness was about how easy it is to clean the cover for the user.^4^ Insurance evaluated if the cost of the cover will get reimbursed or not, by an insurance company. This criterion was directly related and was equally important to the “economical” criterion, as if the cover was overpriced, then the chances for compensation by the insurance company were low. Next, the criterion of “protection” assessed the level of protection a cover provided to the expensive prosthetic components, which was an important factor to consider. Second to last, the criterion “clear website instructions”, evaluated how clear were the website instructions given by the company to the user and prosthetist, about the use and maintenance of their cosmetic cover. Lastly, the criterion of “reliability” addressed whether the company or prosthetist could deliver a cosmetic cover that seamlessly fits the user every time.

Once an appropriate set of criteria have been identified, the WillowWood foam cover was selected as the baseline, where all criteria for this design were marked with the letter “S”. WillowWood was the most appropriate candidate to be set as the baseline since this type of design was the most well-known amongst the profession of Prosthetics. Next, all other candidate designs were compared in a pairwise fashion against the baseline for each of the criteria and marked accordingly following the marking rational specified in **Table 1**.^9^

**Table 1:** Marking rational for a Pugh matrix. The overall evaluation was made by adding the “+” and “-” for each design concept.

BASELINE	CANDIDATE DESIGN	SYMBOL USED
S	BETTER	+
S	MUCH BETTER	++
S	WORSE	-
S	MUCH WORSE	--
S	EQUAL	S

To help discriminate the options even more, the criteria have been also weighted. Although there was no change in the rankings after applying the weightings, it was useful in deciding which design elements were the best to include in the new design.

## RESULTS

After the evaluation of the five designs, WillowWood was placed last (**Table 2**). Although the total score was the lowest amongst the other covers, it was equal or better at the criteria of “economical”; “lightweight” and “insurance”; making it the most suitable option for the buyer who requires a low-cost cover with relatively aesthetic appearance. The Alleles design outperformed the rest of the designs in the following criteria: “lightweight”; “maintenance/repair”; “practicality” and “clear website instructions”. Covers produced by Alleles were the most lightweight starting from 150g, where other covers started from 250g or more (250g>x>700g). The WillowWood foam covers weighted approximately the same as the Alleles design but scored negatively and lowest in most of the other criteria and therefore is considered the least appropriate. Alleles also provided the ability to prosthetists to adjust or modify the shape of the cover after delivery, with the use of a heat gun. This option may be very useful in case of any minor damages to the cover or when the prosthetist needs to make major alignment changes to the prosthesis which may affect the cosmetic appearance of the cover. It also provided the most straightforward website instructions for both user and prosthetist on how to fit and take care of the cover after delivery.

**Table 2:** Pugh matrix evaluation.

		DESIGN CONCEPTS					
EVALUATION CRITERIA	WEIGHTINGS OF CRITERIA	WILLOWWOOD FOAM COVER	AQUA-LEG	UNYQ	LIMB-ART	ALLELES	NOVEL DESIGN
COVER FIT/AESTHETICS	5	S	++	++	+	+	++
ECONOMICAL	5	S	−	−	−	−	−
LIGHTWEIGHT	2	s	−	−	−	S	S
DURABILITY	4	S	+	++	++	++	++
MAINTENANCE/REPAIR	3	S	+	+	+	++	++
EASE OF ATTACHMENT	2	s	s	++	+	+	++
PRACTICALITY	3	S	S	+	+	++	++
CUSTOMISATION	4	S	+	++	++	++	++
EASE OF CLEARNESS	3	S	++	++	++	++	++
INSURANCE	3	S	−	−	S	−	−
PROTECTION	2	S	+	++	++	++	++
CLEAR WEBSITE INSTRUCTIONS	3	S	−	S	−	+	+
RELIABILITY	4	S	++	++	S	+	++
**TOTAL +**		0	10	16	12	16	19
**TOTAL −**		0	7	5	3	3	3
**TOTAL SCORE**		**0**	**3**	**11**	**9**	**13**	**16**
**WEIGHTED TOTAL +**		0	37	54	39	52	63
**WEIGHTED TOTAL −**		0	23	17	10	13	13
**WEIGHTED SCORE**		**0**	**14**	**37**	**29**	**39**	**50**

## DISCUSSION

The three main manufacture methods and five well-known companies that produce commercially cosmetic covers were identified. After a thorough description of each company's cosmetic cover, a pairwise comparison was conducted using a Pugh matrix based on the criteria defined by the researcher. The results of the Pugh matrix let to consider a novel design utilising the best performing criteria from the other candidate designs. The new design concept revealed the opportunity of an improved design by pointing out some of the key design elements that could be included in a new design. In addition, the new design scored a much higher total score when compared to the second-best design (Alleles), hence identifying once again the improvement possibilities such cover design may have in the future.

### Novel design

Following the application of the weightings (**Table 2**), the two leading design concepts, UNYQ and Alleles, scored almost the same. Although both designs had almost an identical total score, the two designs outperformed each other in different design elements, which let to consider creating a new and superior design.

Combining the most appropriate design elements together in a novel design, resulted to a higher total score (50 points) when compared to all the other design concepts (**Table 2**). This is a theoretical novel design but considering that these companies already produced and tested their covers, it can be assumed that the production of such design is feasible. The novel design was described in the next section while following a logical order from **Table 2**.

Starting with the criterion of “cover fit/aesthetics”; 3D scanning technology should be used to capture the true image of the patient's prosthesis and sound leg to establish a perfect fit for the cover. The cost of the cover should be as affordable as possible, but possibly a high price tag is inevitable as the most advanced materials and scanning techniques are meant to be used in the production of such design. An “attractive” price range for the novel design would be between $500 and $1000, although this is influenced by many factors during the production phase. Reducing the cost of the cover will increase the possibilities for the insurance companies to reimburse the cover and may attract more users to buy and try the cover. Unfortunately, no previous studies nor web resources have stated an absolute range of prices an insurance company would reimburse. Ideally the cover should be lightweight and still durable enough to resist impacts and protect the prosthetic componentry, which can be achieved using the MJF 3D printing method and high-grade nylon powder as the material of the cover. In cases of any major damages to the cover, a replacement policy should be provided to the users. Moreover, one of the attachment methods should be with magnets as this may be the fastest way to don and doff the cover. For the criterion of “practicality”, the cover should be able to be “fine-tuned” to any alignment changes made on the prosthesis which may affect the overall shape; this may be achieved with the use of a heat gun, or with a “flexible” cover design consisting of different moving parts or auxetic structures allowing the cover to adjust in any alignment and volume changes. The cover's design and colours should be fully customisable to match the patient's needs.

### Pugh matrix evaluation

To better appreciate the rationale behind the score of each design concept, the first three rows of **Table 2** have been briefly explained.

Beginning with the first row which refers to the criterion of “cover fit/aesthetics”. The WillowWood foam cover, which was set as the baseline, can be described as un-cosmetic around the knee joint, and can change shape over time; in addition, foam covers may not always represent a true mirrored image of the sound leg. Based on the performance of the baseline, UNYQ and Aqua-Leg scored “++”, as their covers perfectly fit on the prosthesis due to the 3D scanning technology used, where the margin of error is minimal. Lastly, Limb-Art and Alleles scored “+”, as they both use manual measuring procedures to manufacture their covers, but they still produce more aesthetic covers than the baseline.

Moving on to the second and third rows, which refer to the criteria of “economical” and “lightweight”. The WillowWood foam cover cost less than $100 and weighs around 150g.^11^ For the “economical” criterion, all other candidates scored worse than the baseline; for instance: Limb-Art scored “-” because their cheaper cover starts from $270^12^ and UNYQ scored “--” because their cheaper cover starts from $495.^13^ For the “lightweight” criterion, all the covers, except Alleles, scored worse than the baseline; for instance: UNYQ scored “--” because their lighter cover weighs 300g,^14^ which was heavier than the baseline; thus, the negative score. The most lightweight Alleles cover weighs 150g,^15^ which was approximately the same as the baseline; therefore, the letter “S” was used.

### Study limitations

The information for each company's cosmetic cover were mainly gathered from their websites which may have influenced the data's reliability as it may be biased in some sections; this approach was used due to lack of available published literature about each cover.

Whilst the criteria set on this study were thought to be inclusive of the main factors which should be considered in the design of a cosmetic cover, they are not exhaustive. It is feasible that alternative criteria could be selected and prioritised differently by different researchers.

### Future research

The criteria and results of the Pugh matrix were based on information found through available literature and websites, making the overall approach of this study to be more towards the objective side rather than subjective. A future study could have end-user groups evaluate the existing list of criteria and even expand the list further, on topics such as reimbursement. Such study may provide sufficient subjective feedback from the users themselves and offer reliable evidence about the different cosmetic options, thus allowing higher quality evidence-based studies to take place in the future.

## CONCLUSION

Although a theoretical novel design was successfully presented through the comparison of the different designs, the data of the study were obtained from the literature and websites of these companies, making the results of the study to be more towards the objective side rather than subjective. Researchers are encouraged to conduct more evidence-based studies around cosmetic covers which are oriented more towards the user's point of view thus allowing future research to be more subjective rather than objective.

## CALL TO ACTION

As we navigate through the intricacies of cosmetic covers, the findings from our study emphasize the need for continued exploration and innovation. Now, we call upon researchers, designers, and professionals in the prosthetic industry to seize this opportunity for advancement. Our research has identified key areas where novel designs can make a substantial impact. By incorporating the best- performing criteria from leading companies like UNYQ,^14^ Limb-Art,^15^ Alleles,^16^ WillowWood,^17^ and Aqua-Leg,^18^ we envision a future where prosthetic cosmetic covers not only enhance appearance but also improve functionality and user experience.

This study lays the groundwork for a more subjective approach to future research. We encourage the prosthetic community to delve deeper into the lived experiences and preferences of prosthetic users. By combining quantitative data with qualitative insights, we can create cosmetic covers that not only meet technical standards but also resonate with the diverse and unique identities of individuals.

## DECLARATION OF CONFLICTING INTERESTS

Dr. Anthony McGarry has no conflict of interest in the preparation of this manuscript. Kyriakos Efstathiou is co-founder of OrthoDesigns.

## AUTHORS CONTRIBUTION

Both authors contributed equally to the research and the writing of this manuscript.

## SOURCES OF SUPPORT

None.

## AUTHORS SCIENTIFIC BIOGRAPHY

**Figure:**
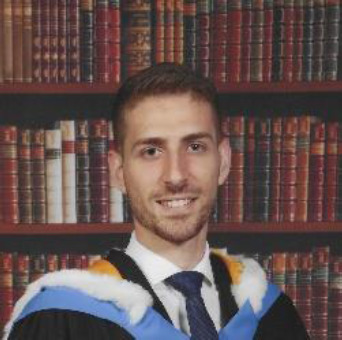


**Kyriakos Efstathiou**, born on June 28, 1999, in Cyprus, discovered the field of Prosthetics and Orthotics (P&O) during a chance meeting with the owner of a local P&O clinic. Motivated by this encounter, he commenced his studies at the University of Strathclyde in 2018, aspiring to become a Certified Prosthetist and Orthotist. During his academic journey, Kyriakos encountered the transformative applications of 3D printing in the P&O field. This revelation ignited his mission to make 3D printing more affordable and accessible to clinics. Upon completing his studies and work placement, Kyriakos returned to Cyprus and co-founded OrthoDesigns alongside Savvas Savva, a P&O Technician. Since then, he has been working as a clinician, contributing locally, while managing OrthoDesigns and steering its expansion both nationally and internationally.

**Figure:**
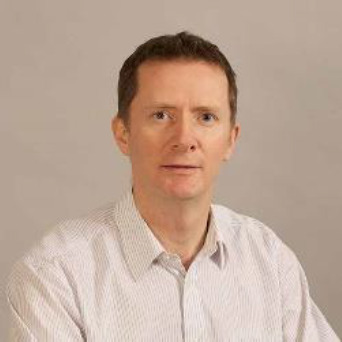


**Dr Anthony McGarry** is a Senior Teaching Fellow, Health Care Professionals Council registered prosthetist/orthotist, Department of Biomedical Engineering, University of Strathclyde. He is responsible for undergraduate/postgraduate tuition in lower limb prosthetics. Interests include: clinical management; socket design, shape capture methods and residual limb interface pressures. He obtained a PhD in Evaluation of prosthetic shape capture systems in the University of Strathclyde in 2009. Current research focus/publication is in evaluating prosthetic shape capture methods and Computer-Aided-Design. For research activity please visit: https://pureportal.strath.ac.uk/en/persons/anthony-mcgarry

## References

[1] Murray CD. Being like everybody else: The personal meanings of being a prosthesis user. Disabil Rehabil. 2009;31(7):573–81. DOI: 10.1080/09638280802240290 19034778

[2] Highsmith MJ, Kahle JT, Knight M, Olk-Szost A, Boyd M, Miro RM. Delivery of cosmetic covers to persons with transtibial and transfemoral amputations in an outpatient prosthetic practice. Prosthet Orthot Int. 2016;40(3):343–9. DOI: 10.1177/0309364614564024 25575552

[3] Fukuda S, editor. Emotional Engineering, Vol. 4. Springer Nature; 2016; DOI: 10.1007/978-3-319-29433-9

[4] Cairns N, Murray K, Corney J, McFadyen A. Satisfaction with cosmesis and priorities for cosmesis design reported by lower limb amputees in the United Kingdom. Prosthet Orthot Int. 2014;38(6):467–73. DOI: 10.1177/0309364613512149 24327666 PMC4230545

[5] Ottobock US shop [Internet]. [cited 2021 Jan 21]. Available from: https://www.ottobockus.com/products/custom-silicone-leg-cover/

[6] Ottobock.nl. [Internet]. [cited 2021 Jan 21]. Available at: https://www.ottobock.nl/media/catalogus/service-fabrication_customized-solutions_en.pdf

[7] Ngo TD, Kashani A, Imbalzano G, Nguyen KTQ, Hui D. Additive manufacturing (3D printing): a review of materials, methods, applications and challenges. Compos B Eng. 2018;143:172–96. DOI: 10.1016/j.compositesb.2018.02.012

[8] Cai C, Tey WS, Chen J, Zhu W, Liu X, Liu T, et al. Comparative study on 3D printing of Polyamide 12 by selective laser sintering and multi jet fusion. J Mater Process Technol. 2021;288:116882. DOI: 10.1016/j.jmatprotec.2020.116882

[9] Burge S. The systems engineering tool box [Internet]. Burgehugheswalsh.co.uk, [cited 2021 Jan 27]. Available from: https://www.burgehugheswalsh.co.uk/uploaded/1/documents/pugh-matrix-v1.1.pdf

[10] Kahle JT, Highsmith MJ. The implications of amputees being overweight [Internet]. In Motion; [cited 2021 Jan 27]. Available from: http://opmarketing.com/storage/Research%20EncyclOPedia/Health/in%20Motion%202008%20amps%20overweight.pdf

[11] BK cosmetic foam cover for prosthetic limbs [Internet]. [cited 2021 Feb 4]. Available from: https://wonderfureha.en.made-in-china.com/product/wyYmFajvSZhG/China-Bk-Cosmetic-Foam-Cover-for-Prosthetic-Limbs.html

[12] Saunders S. Limb-art: Using HP technology to create stylish 3D printed prosthetic leg covers [Internet]. 3dprint.com: The Voice of 3D printing/additive manufacturing; 2021; [cited 2021 Jan 24]. Available from: https://3dprint.com/251688/limb-art-hp-create-3d-printed-prosthetic-leg-covers/

[13] UNYQ raises $1M, taking pre-orders for 3D printed below-knee fairings [Internet]. www.3ders.org; [cited 2021 Jan 25]. Available from: https://www.3ders.org/articles/20140628-unyq-raises-taking-pre-orders-for-3d-printed-below-knee-fairings.html

[14] Invent. Like no other [Internet]. UNYQ; [cited 2021 Jan 22]. Available from: https://unyq.com/

[15] ALLELES, FAQ [Internet]. [cited 2021 Jan 25]. Available from: https://alleles.ca/faq/

[16] Limb-Art Home page [Internet]. [cited 2021 Jan 22]. Available from: https://limb-art.com/about-limb-art/

[17] Transfemoral foam covers [Internet]. WillowWood. [cited 2021 Jan 22]. Available from: https://willowwood.com/products-services/modular-components/cosmetic-foam/transfemoral-foam-covers/

[18] Aqualeg Home page [Internet]. [cited 2021 Jan 22]. Available from: https://www.aqualeg.com/en

